# ﻿*Schiedeahaakoaensis*, a new facultatively autogamous species of ﻿*Schiedea* sect. ﻿*Mononeura* (Caryophyllaceae) from the Hawaiian Islands

**DOI:** 10.3897/phytokeys.210.91226

**Published:** 2022-10-10

**Authors:** Warren L. Wagner, Stephen G. Weller, Ann K. Sakai, Tom DeMent, Josh VanDeMark

**Affiliations:** 1 Department of Botany, MRC-166, National Museum of Natural History, Smithsonian Institution, P.O. Box 37012, Washington, DC 20013-7012, USA National Museum of Natural History, Smithsonian Institution Washington United States of America; 2 Department of Ecology and Evolutionary Biology, University of California, Irvine, CA 92697, USA University of California Irvine United States of America; 3 DLNR/Division of Forestry & Wildlife, 19 E. Kawili St., Hilo, HI 96720, USA DLNR/Division of Forestry & Wildlife Hilo United States of America

**Keywords:** Caryophyllaceae, conservation, Hawaiian Islands, Hawai‘i Island, *Schiedea*

## Abstract

In 2016 during a survey for potential fencing of the Ha‘akoa unit on windward Mauna Kea, Hawai‘i Island (Hawaiian Islands) a single plant of the genus *Schiedea* was discovered. No species of the genus had ever been known to occur in this area, and only three species of *Schiedea* were known previously from Hawai‘i Island. Two are vining species and the third is a coastal subshrub. The single plant obviously represented an interesting find, and because the plant was vegetative another visit was scheduled to collect a flowering specimen, but by then the plant had died. Soil taken from the site with seeds in the soil produced two plants, one of which flowered in cultivation in 2021. A study of this individual indicated it was a member of Schiedeasect.Mononeura, characterized by erect to ascending habit, quadrangular stems, seeds not persistent on the placenta and readily dispersing from the dehisced capsule, and flowers facultatively autogamous. With the discovery of this new species there are 35 species in this Hawaiian endemic genus.

## ﻿Introduction and discussion

An unusual *Schiedea* was discovered by Tom DeMent in September 2016 while scouting the area for a future fence line for the Ha‘akoa Unit. Photos and a leaf sample were taken that day for a second opinion and possible identification. A second visit to the site of the plant was made in July 2017. At that time, only the desiccated remains of the non-reproductive plant were found. The stem was still turgid, the leaves were brown and dry, but still attached. A branch cutting was taken with the hope there was still enough life in the stem to root and resprout. It was unsuccessful. A soil sample was also taken during the 2017 visit, but nothing germinated from it. A third visit took place in 2018 by Tom DeMent, Josh VanDeMark and Reid Loo. The dead individual was completely gone, and the area was surveyed for any new individuals without success. A new soil sample was also taken. Jaime Enoka at the Volcano Rare Plant Facility (**VRPF**) propagated two plants from this soil collection in 2019. One of the plants flowered in 2021, a portion of which was used to make a partial specimen (type), which together with a number of photographic images of the plant in cultivation as well as some of images of the original plant in the field, were used to write the following description.

In our long-term study of the diversification of *Schiedea* Cham. & Schltdl. to distinguish species, especially those clusters of closely related species, we have emphasized changes in breeding systems correlated with discrete floral and vegetative characters (Wagner et al. 2005). Changes in breeding system are usually correlated with habitat and geographical discontinuities. Many species of *Schiedea*, especially those from older islands where extinction of intermediate forms may have occurred, are readily distinguished from all other species (e.g., *S.apokremnos* H. St. John, *S.helleri* Sherff, *S.spergulina* A. Gray, *S.stellarioides* H. Mann, and *S.verticillata* F. Br.). Other species, especially those on younger islands, are less readily separated from their congeners. For example, in sect. Mononeura, *S.laui* W. L. Wagner & Weller was initially viewed as a rediscovery of *S.nuttallii* Hook. on Moloka‘i, albeit in a wetter habitat at higher elevations than is typical for *S.nuttallii* on O‘ahu. Plants grown in the greenhouse, however, showed several morphological differences from *S.nuttallii*. Flowers of all plants of *S.laui* are completely cleistogamous, whereas *S.nuttallii* on O‘ahu has protandrous flowers and is primarily outcrossing (Wagner et al. 2005). Initially, we planned to recognize *S.laui* as a subspecies of *S.nuttallii*, but it failed to group with *S.nuttallii* in phylogenetic analyses using Sanger sequences of several plastid and nuclear loci and morphology (Nepokroeff et al. 2005; Willyard et al. 2011), and therefore we described *S.laui* as a distinct species. In a parallel contrast, *S.jacobii* W. L. Wagner, Weller & Medeiros (Wagner et al. 1999) superficially resembles *S.nuttallii* but occurs allopatrically at higher elevations in very wet forests. Moreover, *S.jacobii* is autogamous and produces seeds that are retained in capsules, a characteristic feature of *Schiedea* species occurring in very wet habitats. Similarly, the new species described here at first seemed very similar to *S.nuttallii* as well as to *S.jacobii* and *S.laui*, but after careful study of all available characters, it possesses a suite of features distinguishing it from these other very similar species, including the erect to ascending habit, quadrangular stems, seeds not persistent on the placenta, readily dispersing from the dehisced capsule, and flowers facultatively autogamous. The molecular studies published so far indicate that *S.nuttallii*, *S.jacobii*, *S.laui*, and *S.kaalae* Wawra are all in the same subclade of sect. Mononeura (Nepokroeff et al. 2005; Willyard et al. 2011). Ongoing analyses using Hyb-Seq target enrichment will include *S.haakoaensis* to resolve more fully the relationships among this group of closely related species of sect. Mononeura that have diversified across all of the main Hawaiian Islands. The morphological, breeding system and geographical/ecological characteristics of the new species and its close relatives are compared in Table 1.

**Table 1.** Comparison of morphological, breeding system, and geographical/ecological characters of the subclade of species of Schiedeasect.Mononeura.

Character	*S.nuttallii*	*S.kaalae*	*S.jacobii*	*S.laui*	*S.haakoaensis*
Stem texture and shape	Weakly fleshy, flattened	Thick and fleshy, rounded, but pedicels flattened	Fleshy, quadrangular, winged	Weakly fleshy, flattened, or upper weakly quadrangular	Fleshy, quadrangular, not winged
Leaf shape	Narrowly ovate or lanceolate to narrowly or broadly elliptic	Elliptic-oblanceolate to nearly spatulate	Lanceolate to oblong-elliptic	Narrowly ovate or lanceolate to narrowly or broadly elliptic	Narrowly or broadly elliptic, sometimes lanceolate to oblanceolate
Leaf length and width (cm)	5–13 × 1.4–3.5	(8–) 14–24 × (1.5–) 2–5 (–6)	4.5–10.5 × 1.4–2.6	6.5–13 × 1.5–2.8	3–18 × 1.2–4.5
Inflorescence length (cm)	Inflorescence with 50–240 flowers, 20–25 (–32) cm long	Inflorescence with 20–300 flowers, 20–40 (–60) cm long	Inflorescence with 10–70 flowers, 40–50 cm long	Inflorescence with 10–18 flowers, 17–26 cm long	Inflorescence with up to 80 or more flowers, 40–50 cm long
Pedicel length (mm) at anthesis	6–12 mm	7–10 mm	3–8 mm	3–11 mm	4–6 mm
Inflorescence pubescence	Bracts ciliate, internodes with scattered hairs	Bracts ciliate	Glabrous	Bracts ciliate, internodes with scattered hairs	Bracts ciliate, internodes with scattered hairs
Pollination	Chasmogamous	Chasmogamous	Facultatively autogamous	Cleistogamous	Apparently facultatively autogamous
Sepal orientation from pedicel	5° to 30°angle	ca. 5° to 10°angle	90° to 135°angle	175° to 180°angle	ca. 30° to 60°angle
Sepal pubescence	Sparsely ciliate	Sparsely ciliate	Glabrous	Sparsely ciliate	Ciliate
Seeds persistent on placenta	No	No	Yes	No	Apparently not
Seed length (mm)	0.9–1	1.0–1.1	0.7–0.9	1.0–1.1	0.8–1.0
Distribution	O‘ahu (Wai‘anae Mts.); previously from Moloka‘i (E end), West Maui	O‘ahu (Wai‘anae and Ko‘olau Mts.)	East Maui (Hanawi)	Moloka‘i (Waikolu)	Hawai‘i (Ha‘akoa unit on windward Mauna Kea)

## ﻿﻿Taxonomic treatment

### ﻿ Schiedea haakoaensis

Taxon classificationPlantaeCaryophyllalesCaryophyllaceae

W. L. Wagner, Weller & A. K. Sakai sp. nov.

25379E4E-4397-5EEC-AC16-741F7CF62F74

urn:lsid:ipni.org:names:77306319-1

Fig. 1

#### Type.

**Hawaiian Islands: Hawai`i**: Hawai‘i County, North Hilo District, Laupāhoehoe Section of Hilo Forest Reserve, windward Mauna Kea, Mauka of Ha‘akoa and Pāhale Streams, 5020 ft (1530 m), Cult. material harvested July 2021, *Tom DeMent & Josh VanDeMark s.n.* (holotype: US-3742219!).

**Figure 1.**
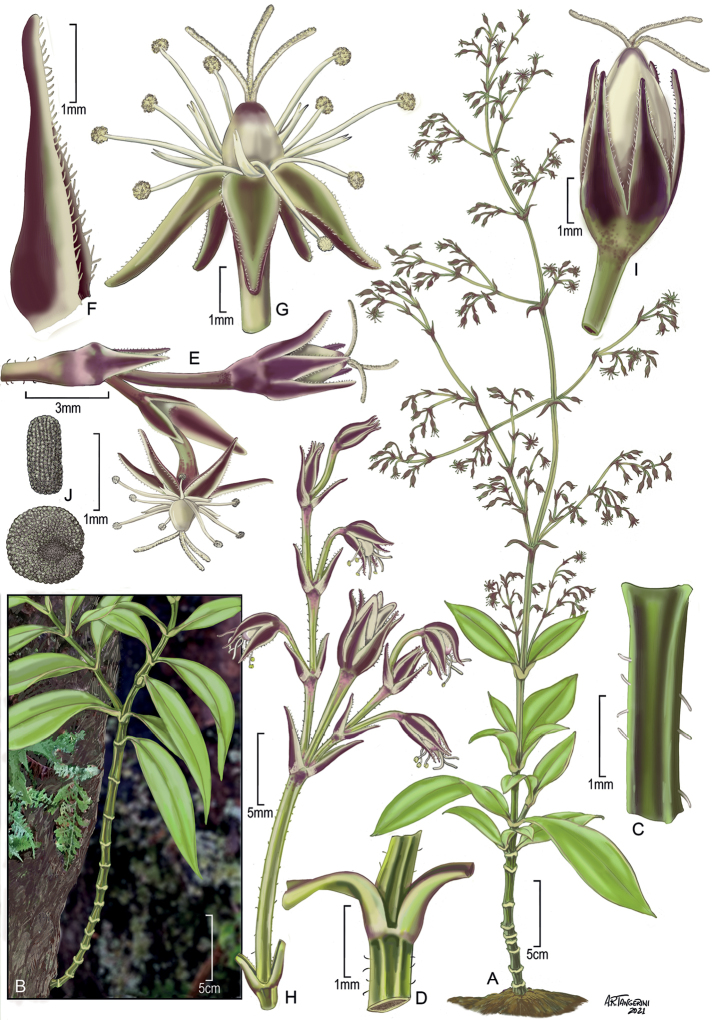
*Schiedeahaakoaensis* W. L. Wagner, Weller & A. K. Sakai **A** habit, stem with leaves and inflorescence **B** habit of plant in habitat **C** stem internode, showing sparse hairs **D** stem internode with leaf bases **E** branch of inflorescence with developing fruit and flower **F** bract **G** flower at full anthesis **H** branch of inflorescence, post anthesis **I** capsule **J** seed. Drawn from photograph of cultivated individual from which holotype was taken (**A**), drawn from field photograph and cropped to fit plate (**B**), drawn from holotype (**C, D, H, I, J**), and from the holotype and photographs (**E, F, G**). Coloration added to plate figures from photographs. Illustration by Alice Tangerini.

#### Description.

Erect to strongly ascending subshrubs 7–10 dm tall or in wild perhaps more; stem single or sometimes with few short side branches, conspicuously quadrangular, pale green, but distal internodes often purple-tinged, glabrous except internodes of inflorescence, bracts and sepals. Leaves opposite; blades 3–18 cm long, 1.2–4.5 cm wide, narrowly or broadly elliptic, sometimes lanceolate to oblanceolate, pale green to yellowish green, sometimes younger ones purple-tinged, slightly thickened and rubbery, chartaceous when dry, with only the midvein evident, the midvein ± slightly excentric, margin entire, slightly thickened becoming weakly involute toward the apex, apex acute to acuminate; petioles 0.5–1 cm long, weakly grooved. Inflorescence terminal, 40–50 cm long, with up to 80 or more flowers, diffuse, erect, the lateral branches 11–18 cm long, ascending, each with 6–20 flowers, the tertiary and higher level internodes, usually ascending or appressed, with pedicels usually spreading at anthesis, sometimes with a few minute curved hairs along the inflorescence internodes; bracts usually yellowish green, foliaceous, and nearly as large as the leaves in the lowest portions of the central axis, those in the upper part of the inflorescence and subtending the flowers, subulate, purple and usually yellowish green near base, margins ciliate; pedicels 4–6 mm long at anthesis, elongating slightly in fruit, conspicuously asymmetrically flattened and weakly quadrangular, sometimes with a few hairs toward the base. Flowers hermaphroditic, facultatively autogamous. Sepals 3–4.1 mm long, lanceolate, purple, and sometimes greenish toward the base, concave to shallowly navicular toward the apex, oriented at ca. 30° to 60°angle to the pedicel, abaxial side smooth and rounded, glabrous, margins weakly scarious, ciliate, apex attenuate. Nectary shaft 1.5–2.1 mm long, gently recurved, apex weakly bifid. Stamens 10; filaments weakly dimorphic, the antisepalous whorl 2.5–3.1 mm long, the alternate whorl 1.5–2.6 mm long; anthers ca. 0.3 mm long, yellow, apparently dehiscing after flower opens. Styles 3, stigmas elongated and apparently receptive when flower opens, and anthers are dehiscing. Capsules 4.3–5 mm long, ovoid. Seeds 0.8–1.0 mm long, suborbicular, slightly asymmetrical, compressed, brown, the surface rugose. Chromosome number unknown.

#### Distribution.

*Schiedeahaakoaensis* was known from only a single plant from Hawaiian Islands, on the northeastern side of Hawai‘i Island, in the Laupāhoehoe Section of Hilo Forest Reserve. No individuals are currently known from the wild but progeny from one of the two individuals grown from seed collected at the type locality are in cultivation at locations in Hilo and Irvine, California, with outplantings anticipated soon.

#### Habitat.

The only known plant of *Schiedeahaakoaensis* was discovered in 2016 in the montane wet forests of windward Mauna Kea and died of natural causes shortly thereafter. Montane wet forests in this area are dominated by closed canopies of *Metrosiderospolymorpha* Gudich. and *Acaciakoa* A. Gray with a sub-canopy comprised of Cheirodendrontrigynum(Gaudich.)A. Hellersubsp.trigynum, *Melicope* sp., *Myrsinelessertiana* A. DC., *Myrsinesandwicensis* A. DC., *Ilexanomola* Hook. & Arn., *Coprosma* sp., *Phytolaccasandwicensis* Endl., and *Vacciniumcalycinum* Sm. The thick and dense vegetation of the understory host *Rubushawaiiensis* A. Gray, *Hydrangeaarguta* (Gaudich.) Y. De Smet & Granados, *Cibotiumglaucum* (Sm.) Hook. & Arn., *Cibotiummenziesii* Hook., *Cibotiumchamissoi* Kaulf., and a rich diversity of additional ferns including Adenophorustamariscinus(Kaulf.)Hook. & Grev.var.tamariscinus, *Aspleniumpolyodon* G. Forst., *Aspleniumaethiopicum* (Burm. f.) Bech., *Athyriummicrophyllum* (Sm.) Alston, *Diplaziumsandwichianum* (C. Presl) Diels, *Dryopteriswallichiana* (Spreng.) Hyl., *Microlepiastrigosa* (Thunb.) C. Presl, PolypodiumpellucidumKaulf.var.pellucidum, *Sadleria* sp., and the uncommon *Aspleniumschizophyllum* C. Chr. The forests of this area have a low number of non-native species, most of which occur in the understory and consist of non-native grasses and shrubs including *Cenchrusclandestinus* (Hochst. ex Chiov.) Morrone, *Ehrhartastipoides* Labill., *Rubusargutus* Link, *Passifloratarminiana* Coppens & Barney and *Juncus* sp. The terrain and topography of the region are carved by seasonal and perennial streams with gulches varying in depth from a few meters to very large river gulches at lower elevations. The area of the previously known individual, ~5000 ft (1525 m) elevation, lies uphill from the dominant streams of Ha‘akoa and Pāhale with a mean annual rainfall of ~115 inches (292 cm; Rainfall Atlas of Hawaii).

#### Threats.

Habitat degradation by feral pigs continues to threaten species that grow at ground level in the understory of these forests, which remain unprotected by ungulate-proof exclusionary fences. Currently, a ~1200-acre fenced unit is being constructed in the area and once completed will encompass the area where *Schiedeahaakoaensis* was discovered. The sole individual of *S.haakoaensis* was naturally protected from feral pig damage due to its location on a log elevated 3–4 ft (0.9–1.2 m) above the ground. Although ungulate damage was not the cause of death other threats to extremely rare species exist. The only individual of *S.haakoaensis* was probably affected by a prolonged period of drought and its particular location on the log.

#### Breeding system.

Facultative autogamy is indicated by the occurrence of abundant seed production of the one plant that has so far flowered at the VRPF. The plant is growing in an enclosed facility, but some pollinator access is possible. Most species in the VRPF that have floral adaptations for pollinator attraction do not produce fruits unless pollinated, suggesting that the abundant fruit production of *S.haakoaensis* is likely to result from self-pollination. Nearly all wet forest species of *Schiedea* are facultatively or obligately autogamous with self-pollination and subsequent self-fertilization facilitated by synchrony between stigma receptivity and release of pollen from anthers.

#### Conservation assessment.

IUCN Red List Category. When evaluated using the IUCN criteria for endangerment, *Schiedeahaakoaensis* falls into the Extinct in the Wild (EW) category. Surveys of the surrounding area and habitat have failed to locate additional individuals although it is possible more exist in the vast and remote landscape of the windward Mauna Kea wet forests. Future surveys of the area should concentrate on the adjacent US Fish & Wildlife Maulua Tract, the Laupāhoehoe Section of Hilo Forest Reserve, and the Laupāhoehoe Natural Area Reserve. Future protection and feral ungulate exclusion in these areas will help preserve habitat for this species should it be rediscovered, and for individuals of *S.haakoaensis* restored to the area.

## Supplementary Material

XML Treatment for Schiedea haakoaensis
